# Fe_3_O_4_ Nanoparticles Loaded on Lignin Nanoparticles Applied as a Peroxidase Mimic for the Sensitively Colorimetric Detection of H_2_O_2_

**DOI:** 10.3390/nano9020210

**Published:** 2019-02-06

**Authors:** Qingtong Zhang, Mingfu Li, Chenyan Guo, Zhuan Jia, Guangcong Wan, Shuangfei Wang, Douyong Min

**Affiliations:** 1 College of Light Industry and Food Engineering, Guangxi University, Nanning 530004, China; qingyutong110@163.com (Q.Z.); mingfuli@mail.gxu.cn (M.L.); guochenyan2017@163.com (C.G.); jiazhuan123@163.com (Z.J.); wanguangcong@163.com (G.W.); wangsf@gxu.edu.cn (S.W.); 2 Guangxi Key Lab of Clean Pulp & Papermaking and pollution Control, Nanning 530004, China

**Keywords:** Fe_3_O_4_ nanoparticles, Lignin nanoparticles, peroxidase mimic, colorimetric, H_2_O_2_ detection

## Abstract

Lignin is the second largest naturally renewable resource and is primarily a by-product of the pulp and paper industry; however, its inefficient use presents a challenge. In this work, Fe_3_O_4_ nanoparticles loaded on lignin nanoparticles (Fe_3_O_4_@LNPs) were prepared by the self-assembly method and it possessed an enhanced peroxidase-like activity. Fe_3_O_4_@LNPs catalyzed the oxidation of 3,3′,5,5′-tetramethylbenzidine (TMB) in the presence of H_2_O_2_ to generate a blue color, was observable by the naked eye. Under the optimal conditions, Fe_3_O_4_@LNPs showed the ability of sensitive colorimetric detection of H_2_O_2_within a range of 5–100 μM and the limit of detection was 2 μM. The high catalytic activity of Fe_3_O_4_@LNPs allows its prospective use in a wide variety of applications, including clinical diagnosis, food safety, and environmental monitoring.

## 1. Introduction

Hydrogen peroxide (H_2_O_2_), as one of the most important reactive oxygen species (ROS), is widely used in the food and chemical industries, clinical medicine, and environmental monitoring [1,2,3]. Studies have shown that long-term exposure to H_2_O_2_ can cause serious harm to a person’s health, including poisoning, respiratory inflammation [4], cancer, Alzheimer’s disease, and Parkinson’s disease [5]. Therefore, the substantial research efforts are devoted to develop methods for the detection of H_2_O_2_,such ashigh-performance liquid chromatography (HPLC) [6], electrochemistry [1], spectrometry [7], chemiluminescence [8], and colorimetric assays [9]. HPLC, electrochemistry, and chemiluminescence methods are sensitive, but their processes are complex and time-consuming. Spectrometry has the advantage of a simple and low-cost operation but exhibits the disadvantage of poor sensitivity. At present, colorimetric assay methods have been garnering attention due to their high sensitivity and simplicity. Furthermore, colorimetric assay results can be observed unaided and without the use of any sophisticated instruments. Horseradish peroxidase (HRP) is commonly used as a catalyst in conventional colorimetric assay methods to obtain the color signal. As a natural enzyme, HRP is easily affected by acidity, temperature, and inhibitors. In addition, the high cost associated with its preparation, purification and extreme storage conditions also inevitably limits its widespread application [10].

Since the first report of Fe_3_O_4_ nanoparticles (Fe_3_O_4_ NPs) possessing peroxidase-like activity [11], many other nanomaterials, including CeO_2_ nanoparticles [12], NiO_2_ nanoparticles [13] and MnO_2_ nanosheets [14], have also demonstrated intrinsic peroxidase-like activity. These metal nanomaterials are low-cost, sensitive, and maintain their activities in a relatively wide range of environmental conditions, but they all present the problem of agglomeration [15]. To tackle this problem, researchers use graphene or graphene oxide as a structural support to prevent these metal nanomaterials from aggregating in solution [16,17]. However, the high cost of graphene and graphene oxide seriously restricts the widespread application of metal nanozymes.

**Figure 1:**
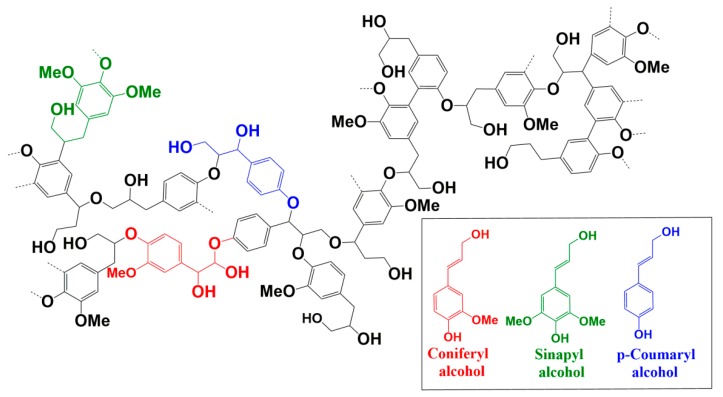
The proposed structure of lignin. Inset: three primary units of lignin.

Lignin is the second most abundant organic macromolecular compound from biomass after cellulose [18]. The primary structure of lignin is composed of three different phenylpropane monomer units: guaiacyl (G), syringyl (S), and p–hydroxyphenol (H) units [19]. These monomer units are mainly linked by ether bonds (β-O-4, 4-O-5, α-O-4, α-O-γ, etc.) and carbon-carbon bonds (β-β, β-1, β-5, 5-5, β-6, etc.) [20,21]. A proposed structural diagram of lignin is shown in Figure 1. Similar to graphene oxide, lignin molecules contain many functional groups, such as hydroxyl and carboxyl groups, which offers the possibility of a stable dispersion of lignin-based materials in aqueous solutions. Recent studies showed that lignin-based nanoparticles can disperse stably in aqueous solutions [18]. Therefore, metal nanozymes carried by lignin nanoparticles can be stably dispersed in solution. Nevertheless, the literature contains few reports of lignin nanoparticles being used to enhance the dispersity of metal nanozymes in the colorimetric detection of H_2_O_2_.

As a common mimetic enzyme of horseradish peroxidase, Fe_3_O_4_ NPs have high catalytic activity and stability during reactions [22]. However, Fe_3_O_4_ NPs also present the urgent problem of agglomeration in solution. In this work, we explored the influence of the preparation conditions on the morphology of Fe_3_O_4_@LNPs. Then, Fe_3_O_4_ NPs loaded on lignin nanoparticles (Fe_3_O_4_@LNPs) were prepared. The prepared Fe_3_O_4_@LNPs exhibited excellent dispersion stability in water and showed higher catalytic activity than Fe_3_O_4_ NPs. The prepared Fe_3_O_4_@LNPs effectively catalyzed H_2_O_2_ to generate hydroxyl radicals, followed by the oxidation of 3,3′,5,5′-tetramethylbenzidine (TMB) [23]. The oxidized TMB had a characteristic absorbance at 652 nm [24]. Therefore, in the presence of H_2_O_2_, TMB, and Fe_3_O_4_@LNPs as catalyst, the reaction system changed from colorless to blue that was identified by the naked eye or quantified by UV-vis spectrophotometer. Because of its excellent dispersion stability in aqueous solution and sensitive peroxidase-like catalytic activity, Fe_3_O_4_@LNPs can be used in a diverse set of applications.

## 2. Experimental

### 2.1. Materials and Reagents

Spruce chips were obtained from a Swedish farm. Tetrahydrofuran (THF), Fe_3_O_4_ NPs (average diameters: 20–30 nm), and TMB were purchased from Aladdin Industrial Co., Ltd. (Shanghai, China). The other analytical grade chemicals were purchased from Boyu Chemicals Company (Nanning, Guangxi, China), and were used without further purification. 

The chips were cooked in a pressure vessel under the following conditions: the total titratable alkali value was 26%, the sulfidity was 20%, the solid-to-liquid ratio was 1:4, a residence time was 4.5 h, and the temperature was 165 °C. When the cooking was completed, the separated black liquor was adjusted to pH = 2 with 2 M H_2_SO_4_ while stirring to precipitate lignin. After standing for 3 h, the supernatant was removed. After centrifugation, washing, and freeze-drying, the crude Kraft lignin was obtained. The crude Kraft lignin was dissolved in THF and stirred for 30 min; the undissolved material was removed by filtering through a 0.45 μm organic filter membrane (Pall Scientific, New York, NY, USA). The lignin/THF solution was stirred under the fume hood until the THF evaporated completely. Then, the powder was dried at 40 °C under vacuum for 48 h to obtain the purified Kraft lignin. 

### 2.2. Statistical Analysis 

All the experiments were performed in triplicate, and the averaged values were reported in this study.

### 2.3. Preparation of Fe_3_O_4_@LNPs

The purified lignin was dissolved in THF to prepare solutions of 0.5, 1.0, and 5.0, mg/mL. With vigorous mechanical stirring, 0.1 mg/mL Fe_3_O_4_ NPs suspension was added dropwise to the lignin/THF solutions using a microsyringe until the suspension with 20% consistency was obtained. The suspension was stirred for an additional 20 min to evaporate THF. Then, the suspension was transferred into a dialysis bag (MWCO: 6000, Union Carbide, Connecticut, CT, USA) and immersed in an excess of deionized water to remove the residual THF. Little trace of THF was detected after 24 h dialysis. Then, the suspension was centrifuged to achieve the solid part which was freeze-dried to obtain Fe_3_O_4_@LNPs. Fe_3_O_4_@LNPs prepared from the solution with 0.5, 1.0, and 5.0 mg/mL initial lignin concentration was respectively denoted as L_1_, L_2_, and L_3_.

### 2.4. Peroxidase-Like Activity of Fe_3_O_4_@LNPs

The peroxidase-like activity of Fe_3_O_4_@LNPs was investigated by catalytically oxidizing TMB in the presence of H_2_O_2._The concentrations of TMB and H_2_O_2_were respectively 4 mM and 100 mM, 0.1 M Citric acid-disodium hydrogen phosphate solution (CPBS) (pH = 3.0) was applied as the buffer, and the final volume of the mixture was 3 mL. The mixture was incubated at 50 °C for 30 min. As for the blank experiments, Fe_3_O_4_@LNPs was replaced by LNPs (lignin nanoparticles) or Fe_3_O_4_ NPs to complete the reaction under the same conditions. The oxidized TMB had a maximum absorbance at 652 nm [25]. Therefore, the peroxidase-like activity of Fe_3_O_4_@LNPs was evaluated through changes of the solution color and the absorbance at 652 nm.

### 2.5. Steady Kinetic Analysis of Fe_3_O_4_@LNPs

The steady-state kinetic measurements were carried out to investigate the mechanism of the peroxidase-like activity of Fe_3_O_4_@LNPs at room temperature. Approximately 600 μL of Fe_3_O_4_@LNPs (264 μg/mL) was mixed with 1.8 mL CPBS (0.1 M, pH = 3.0). As for the first experiment, 300 μL H_2_O_2_ (100 mM) was used, while the TMB concentration was varied (0.4, 0.6, 0.8, 1.0, 1.5, 2.0, 3.0, 4.0 and 6.0 mM). As for the second experiment, 300 μL TMB (4.0 mM) was used, while the concentration of H_2_O_2_ was varied (10, 15, 25, 30, 50, 100, 150, and 250 mM). TheMichaelis-Menten constant was calculated from the Lineweaver-Burk double reciprocal plot by the following equation: 1/ν = K_m_/ν_m_(1/[S] + 1/K_m_), where ν is the initial velocity, K_m_ is the Michaelis constant, ν_m_ is the maximum reaction rate, and [S] is the concentration of substrate [26].

### 2.6. Colorimetric Detection of H_2_O_2_

For the colorimetric analysis of H_2_O_2_, 600 μL Fe_3_O_4_@LNPs (264 μg/mL), 300 μL of TMB (4 mM) and 300 μL H_2_O_2_ (different concentrations) were mixed with 1.8 mL CPBS (0.1 M, pH = 3.0). The mixed solution was incubated at 50 °C for 30 min. The absorbance of the resulted sample measured by a UV-visible spectrophotometer at 652 nm was used to generate the standard curve for the detection of H_2_O_2_.

The limit of detection (LOD) of H_2_O_2_ was calculated based on previous literature [27] via the following equation: LOD = 3S/M, where S is the standard deviation of blank samples and M is the slope of the linear curve between the absorbance at 652 nm and H_2_O_2_ concentration. Eleven blank experiments were carried out as follows: 600 μL Fe_3_O_4_@LNPs (264 μg/mL), 300 μL of TMB (4 mM) and the corresponding volume (300 μL) of H_2_O replacing H_2_O_2_ were mixed with 1.8 mL CPBS (0.1 M, pH = 3.0). The mixtures were incubated at 50 °C for 30 min. The absorbance of 11 blank experiments at 652 nm was collected to calculate S value.

### 2.7. Characterization

The ^31^Pnuclear magnetic resonance (NMR) spectrum of lignin was acquired on an NMR spectrometer (AVANCE III 500 MHz, Bruker, Germany). The morphologies and element analysis of Fe_3_O_4_@LNPs were recorded by field emission transmission electron microscope (FE-TEM) (Tecnail G2F20, FEI Company, Hillsboro, OR, USA) with an accelerating voltage of 200 KV. UV-vis spectra were recorded with a UV-visible spectrophotometer (Agilent 8453, California, CA, USA). The size and zeta potential of the samples were analyzed with a Nanoparticle Size analyzer (Nano-ZS90X, Malvern, Malvern, UK).

## 3. Results and Discussion

### 3.1. Quantification of Hydroxyl Groups of Lignin 

**Figure 2:**
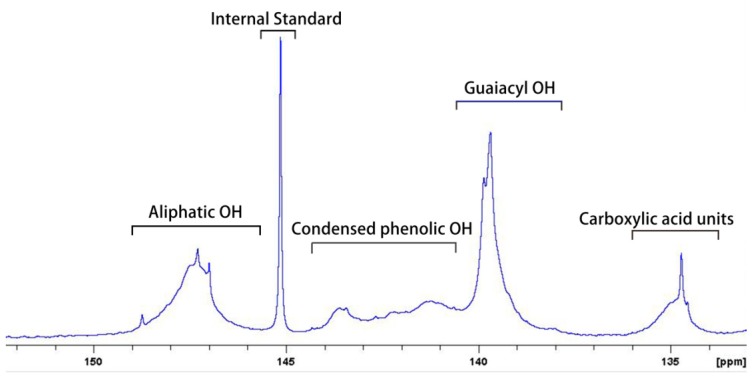
^31^P NMR spectra of the purified lignin.

The hydroxyl groups of lignin was quantified by ^31^P NMR according to the reported method [28]. Approximately 20 mg of lignin was placed in a 1 mL vial. Then, 500 μL of a prepared solution of anhydrous pyridine and CDCl_3_ (1.6:1, *v*/*v*) was added and magnetically stirred. Subsequently, 100 μL of chromium (III) acetylacetonate (5 mg/mL, dissolved in pyridine/CDCl_3_, 1.6:1, *v*/*v*) was added as a relaxation agent and 100 μL of cyclohexanol (30 mg/mL) was added as an internal standard. Finally, 85 μL of phosphitylation reagents was added. After stirring for 10 min, the resulting solution was transferred to a 5 mm NMR tube and tested by NMR spectrometer. The ^31^P NMR spectrum of lignin was shown in Figure 2. The result indicated that the hydroxyl group of guaiacylwas the most abundant in the lignin that is 3.76 mmol/g based on lignin, followed by the aliphatic hydroxyl that was 2.82 mmol/g, the condensed phenolic hydroxyl that was 1.90 mmol/g, and the carboxyl group that was 0.63 mmol/g. More hydroxyl groups indicated the lignin nanoparticles dispersed more stably in water [18]. 

### 3.2. Optimization of Fe_3_O_4_@LNPs Formation

**Figure 3:**
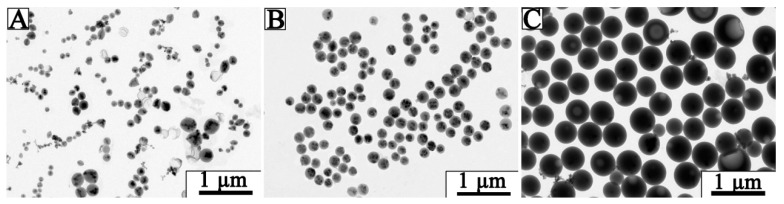
TEM images of Fe_3_O_4_@LNPs prepared by (**A**) L_1_: 0.5 mg/mL, (**B**) L_2_: 1.0 mg/mL, and (**C**) L_3_: 5.0 mg/mL lignin.

The lignin hardly solubilized in water but dissolved in some organic solvents (e.g., THF). Therefore, Fe_3_O_4_NPs suspension used as the anti-solvent and THF used as the solvent were applied to prepare Fe_3_O_4_@LNPs through the lignin self-assembly. The initial concentration of lignin played a vital role in the morphology of lignin nanoparticles [29]. Figure 3 shows that Fe_3_O_4_@LNPs exhibited spherical shape. However, the size of Fe_3_O_4_@LNPs and its distribution varied with the initial concentration of lignin. Compared to L_1_ prepared from 0.5 mg/mL lignin solution, L_2_ and L_3_ respectively prepared from 1.0 mg/mL and 5.0 mg/mL were more homogenous. Furthermore, L_2_ had the highest load of Fe_3_O_4_ NPs. 

**Figure 4:**
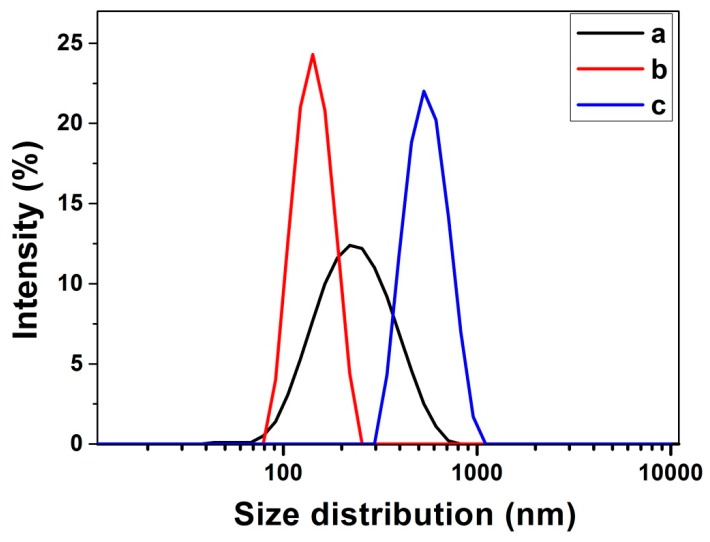
Size distribution of Fe_3_O_4_@LNPs prepared by (**a**) L_1_: 0.5 mg/mL, (**b**) L_2_: 1.0 mg/mL, and (**c**) L_3_: 5mg/mL lignin.

The effect of lignin concentrations on the size and the size distribution of Fe_3_O_4_@LNPs were analyzed bydynamic light scattering (DLS). Figure 4 shows L_2_ possessed a narrow size distribution with an average size of 152.8 nm. Compared to L_2_, L_1_ possessed a wide size distribution with an average size of 181.8 nm, and L_3_ possessed a wide size distribution with an average size of 764.0 nm. The result indicated that 1.0 mg/mL was the optimum initial concentration of lignin for Fe_3_O_4_@LNPs preparation.

In the process of adding water droplets to the lignin/THF solution, lignin as an amphiphilic polymer, with its hydrophilic and hydrophobic properties, gradually aggregates and forms particles, which comprises the “nucleation growth mechanism” [18]. Figure 3 demonstrates that the morphology of the formed nanoparticle was impacted by the initial concentration of lignin. For example, 0.5 mg/mL lignin was applied, the average diameter of L_1_ was only 181.8 nm but the size distribution was wide. The amount of lignin hardly meets the demand for the nanoparticles growth, resulting in the heterogeneous Fe_3_O_4_@LNPs (Figure 3A). However, when the high initial lignin concentration was applied, the extra lignin continued to aggregate on the surface of the formed nanoparticles, eventually leading to the large size Fe_3_O_4_@LNPs (e.g., L_3_) (Figure 3C). The homogeneous morphology of L_2_ indicated that 1.0 mg/mL was the optimum concentration for Fe_3_O_4_@LNPs preparation (Figure 3B). The result was consistent with the previous report [30].

The surface charge of Fe_3_O_4_@LNPs was evaluated by the zeta potential. The results indicated that L_1_, L_2_, and L_3_ possessed comparable negative zeta potentials of −29.2 mV, −32.4 mV and −32.4 mV, respectively, indicating that the initial lignin concentration had little effects on the surface charge of Fe_3_O_4_@LNPs. Therefore, L_1_, L_2_, and L_3_ dispersed stably in aqueous solutions for an extended time without aggregation due to the electrical double layer repulsion induced from the protonation of phenolic hydroxyl and carboxyl groups of lignin. The comparable result was reported that LNPs dispersed stably in water for two months [18]. Based on the above results, L_2_ was applied as the starting material for the further analysis in this study.

### 3.3. Characterization of Fe_3_O_4_ @LNPs

Fe_3_O_4_@LNPs surface possessed the negative charge which was explained as follows. The abundant hydroxyl and carboxyl groups of LNPs provide Fe_3_O_4_@LNPs surface a negative charge. In addition, when the hydrophobic surface of LNPs contacts with water, the hydroxyl ions are absorbed by the hydrophobic surface of LNPs, resulting the generation of strong negative zeta potential on the surface of Fe_3_O_4_@LNPs [18]. Therefore, Fe_3_O_4_ NPs loaded on LNPs can stabilize Fe_3_O_4_ NPs and prevent the aggregation of Fe_3_O_4_ NPs in solution.

**Figure 5:**
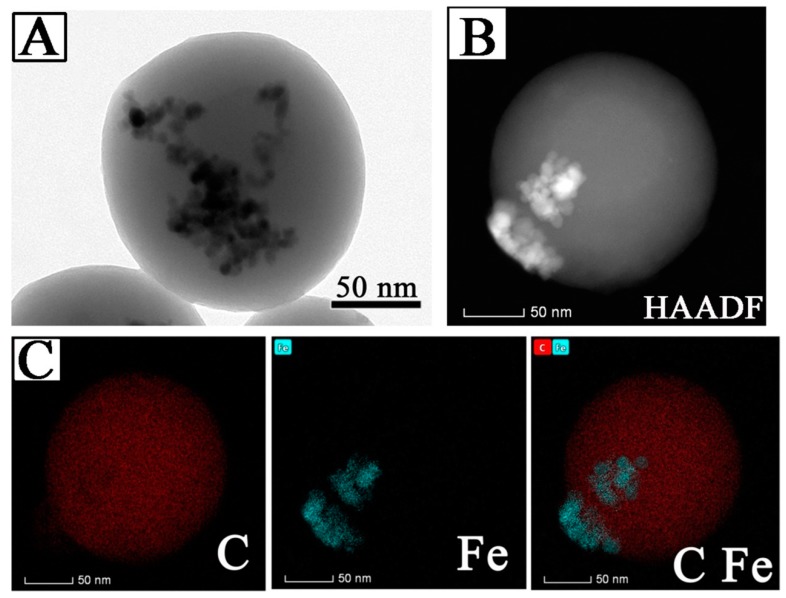
(**A**) HRTEM image of individual Fe_3_O_4_@LNPs; (**B**) High-angle annular dark field image (HAADF) and (**C**) element mapping analysis of Fe_3_O_4_@LNPs.

**Figure 6:**
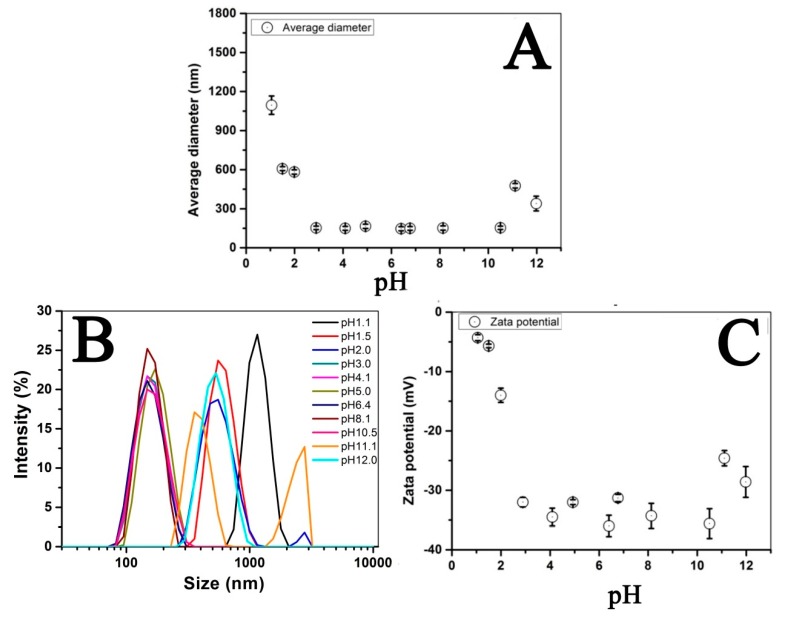
Effects of pH on (**A**) average size of Fe_3_O_4_@LNPs, (**B**) size distribution of Fe_3_O_4_@LNPs, and (**C**) zeta potential value of Fe_3_O_4_@LNPs.

The high resolution transmission electron microscope (HRTEM) image of individual Fe_3_O_4_@LNPs confirmed that Fe_3_O_4_NPs was loaded on the surface of LNPs (Figure 5A). The high-angle annular dark field (HAADF) image demonstrated the morphology of individual Fe_3_O_4_@LNPs, where C and Fe element respectively stands for LNPs and Fe_3_O_4_ NPs (Figure 5B,C). Thus, the HAADF and element mapping analysis confirmed the formation of Fe_3_O_4_@LNPs. The correlation of the size distribution of Fe_3_O_4_@LNPs and pH was investigated by DLS (Figure 6B). The result indicated that Fe_3_O_4_@LNPs had a narrow size distribution within pH from 3.0 to 10.5, confirming that Fe_3_O_4_@LNPs can stably disperse in aqueous solution with a wide pH range. This considerable dispersion ability of Fe_3_O_4_@LNPs was correlated with its zeta potential (approximately −30 mV) in solution within pH 3.0–10.5 (Figure 6C). When pH was below 3.0, the decrease of zeta potential induced the aggregation of Fe_3_O_4_@LNPs. On the other hand, when pH was above 11, the surface charge decreased significantly which caused the aggregation of Fe_3_O_4_@LNPs enlarging the average diameter of nanoparticles. The surface charge of Fe_3_O_4_@LNPsdecreased with the increased concentration of Na^+^ counter ions associated with the addition of NaOH for the pH adjustment. The result was consistent to the reported result on the dispersion stability of polymer nanoparticles correlated with the pH change [18,31].

### 3.4. Optimizing the Reaction Conditions of H_2_O_2_ and TMB Catalyzed by Fe_3_O_4_@LNPs 

**Figure 7:**
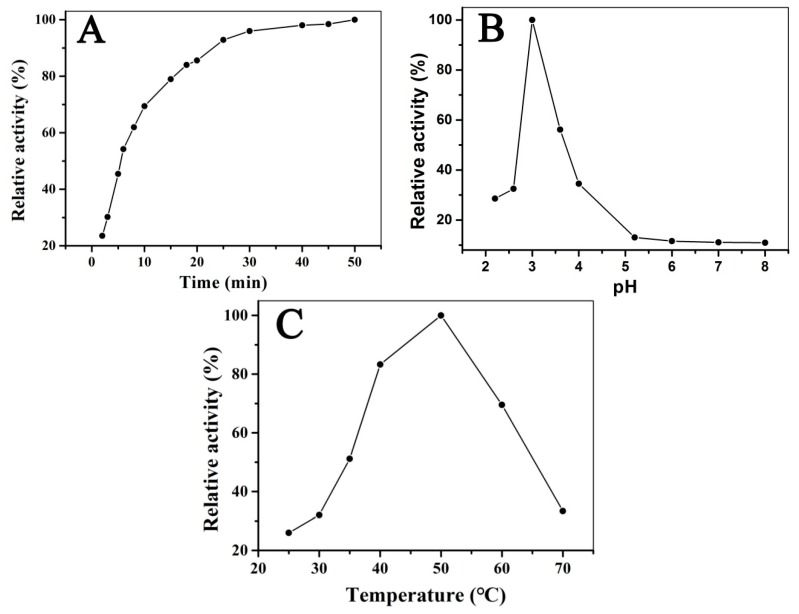
The catalytic activity of Fe_3_O_4_@LNPscorrelated with time (**A**), pH (**B**), and temperature (**C**).

To improve the catalytic activity of Fe_3_O_4_@LNPs, the main parameters, e.g., time, pH, and temperature, were optimized. The experiment was performed as following 600 μL Fe_3_O_4_@LNPs (264 μg/mL), 300 μL TMB (4 mM) and 300 μL H_2_O_2_ (100 mM) were applied as reagents in 1.8 mL CPBS buffer solution (0.1 M). The relative activity of Fe_3_O_4_@LNPs with different pH, time and temperature were defined by follows: the maximum absorbance of the solution after reaction at 652 nm was set as 100%, and the relative activities were calculated by the equation: R = (A/A_max_) × 100%, where R was the relative activity; A was the absorbance of the solution at 652 nm; and A_max_ was the maximum absorbance at 652 nm. The impacts of reaction time, pH, and temperature on the activity of Fe_3_O_4_@LNPs were demonstrated in Figure 7. The correlation between the reactivity of Fe_3_O_4_@LNPs and time was demonstrated in Figure 7A. It was shown that the activity of Fe_3_O_4_@LNPs increased to the maximum within 30 min. Thus, 30 min was set as the optimal reaction time for the following investigations. Within pH = 2.0–4.5, the Fe_3_O_4_@LNPs possessed a high and stable activity and achieved the maximum activity at pH = 3.0 (Figure 7B) that was contributed to solubilization of TMB in the acidic medium [32]. The comparable peroxidase-like activity were reported from HRP and other metal nanoenzymes [10,25]. The catalytic activity was significantly decreased when Fe_3_O_4_@LNPs was applied without the optimal pH (Figure 7B) because of the inherent defect of Fenton reaction [33]. It was reported that the catalytic activity of Fe_3_O_4_NPs loaded on multi-welled carbon nanotubes retained from pH 1–10 [33]; however the high cost of multi-welled carbon nanotubes limited its application. The catalytic activity of Fe_3_O_4_@LNPs was also impacted by temperature, and the maximum activity was achieved at 50 °C (Figure 7C). Compared to natural enzymes and Fe_3_O_4_ nanozymes [11], Fe_3_O_4_@LNPs retained the catalytic activity within a wide temperature range from 30 °C to 60 °C because of its considerable dispersion ability in the aqueous solution.

### 3.5. Peroxidase-liked Activity of Fe_3_O_4_@LNPs

**Figure 8:**
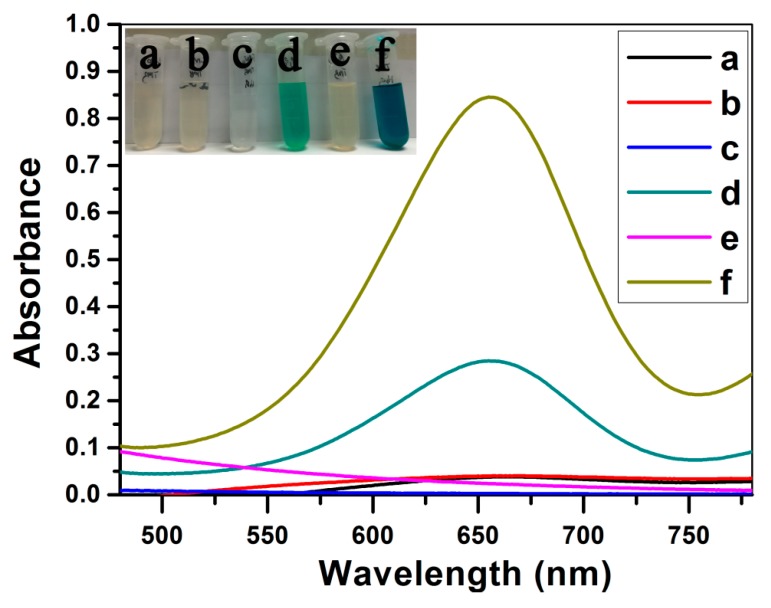
The UV-vis spectra of LNPs + TMB (**a**); LNPs + TMB + H_2_O_2_ (**b**); Fe_3_O_4_ + TMB (**c**); Fe_3_O_4_ + TMB + H_2_O_2_ (**d**); Fe_3_O_4_@LNPs + TMB (**e**) and Fe_3_O_4_@LNPs + TMB+ H_2_O_2_ (**f**); Insert is the photograph of the corresponding solution.

The peroxidase-liked activity of Fe_3_O_4_@LNPs was confirmed and evaluated by the reaction between TMB and H_2_O_2_ under optimal conditions. Little color changes and absorbance peaks at 652 nm were observed from the reaction of LNPs + TMB and LNPs + TMB + H_2_O_2_ (Figure 8 curve a and b), which suggested that LNPs possessed little catalytic activity. Little color change and absorbance peak at 652 nm were observed from the reaction of Fe_3_O_4_ NPs + TMB, confirming that TMB could not be oxidized without the presence of H_2_O_2_. The color change and absorbance peak at 652 nm were respectively observed from the reaction of Fe_3_O_4_ NPs + TMB + H_2_O_2_ and Fe_3_O_4_@LNPs + TMB + H_2_O_2_, which indicated TMB was oxidized during the reaction. However, the absorbance peak of Fe_3_O_4_@LNPs + TMB + H_2_O_2_ at 652 nm was significantly higher than that of Fe_3_O_4_ + TMB + H_2_O_2_, indicating Fe_3_O_4_@LNPs possessed stronger peroxidase-liked catalytic activity than Fe_3_O_4_ NPs due to its considerable dispersion ability. It was consistent with the reported result that Fe_3_O_4_ NPs stabilized by chitosan exhibited strong catalytic activity than Fe_3_O_4_ NPs [34].

### 3.6. Steady-State Kinetic Study of Fe_3_O_4_@LNPs

**Figure 9:**
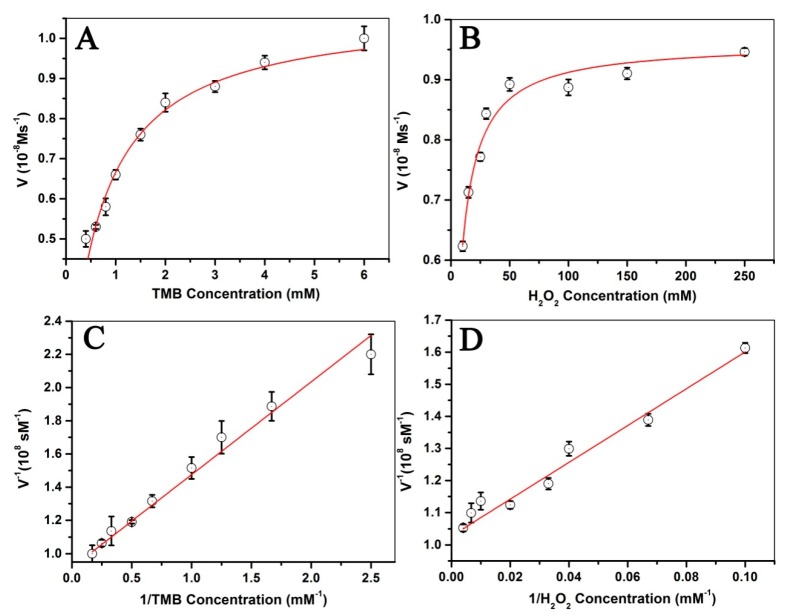
The steady-state dynamic analysis and catalytic mechanism of Fe_3_O_4_@LNPs: the Michaelis-Menten curves (**A**,**B**) and the double reciprocal plots of the activity of Fe_3_O_4_@LNPs(**C**,**D**).

The steady-state kinetic mechanism of Fe_3_O_4_@LNPsNPs reacted with H_2_O_2_ and TMB was investigated. The concentration of TMB-derived oxidation products was calculated by A = ε × b × C (Beer-Lambert Law), where A is the absorbance value at 652 nm at room temperature and the molar absorption coefficient (ε) is 39,000 M^−1^cm^−1^ [35]. The Michaelis-Menten curves were obtained by following steps: the reaction rates of Fe_3_O_4_@LNPsNPs in different concentration of TMB or H_2_O_2_ were obtained by calculated the slope of the reaction system at 652 nm in the initial 5 min. Then, the reaction rates were plotted on the ordinate and the concentrations of TMB or H_2_O_2_ were plotted on the abscissa. The Lineweaver-Burk plots were obtained by taking the reciprocal of the reaction rate as the ordinate and the reciprocal of the concentration of TMB or H_2_O_2_. The reaction rate correlated with TMB concentration and H_2_O_2_ concentration was respectively demonstrated in Figure 9A,B. The Michaelis-Menten constant (K_m_) is considered to be a key criterion for evaluating the enzyme’s affinity to a substrate [32]. The maximum velocity (V_m_) and K_m_ were obtained from the Lineweaver-Burk plots (Figure 9C,D) and summarized in Table 1. The K_m_ values of Fe_3_O_4_@LNPs towards TMB and H_2_O_2_ was comparable to that of HRP which indicated the Fe_3_O_4_@LNPsNPs could be used as an effective substitute for HRP. The K_m_ value of Fe_3_O_4_@LNPsNPs with TMB as the substrate was higher than that of Fe_3_O_4_ NPs with TMB, indicating that the Fe_3_O_4_ NPs have a higher affinity to TMB. The K_m_ value of the Fe_3_O_4_@LNPs with H_2_O_2_ was 29 times lower than that of the Fe_3_O_4_NPs with H_2_O_2_, suggesting that the Fe_3_O_4_@LNPs possessed significantly stronger affinity to H_2_O_2_ than the Fe_3_O_4_ NPs. However, the low K_m_ value of Fe_3_O_4_@LNPswith H_2_O_2_is potentially due to the continuous release of Fe_3_O_4_NPs from the Fe_3_O_4_@LNPs, thus avoiding the aggregation of Fe_3_O_4_ NPs and providing more reactive sites which enhanced the peroxidase-like activity of Fe_3_O_4_@LNPs during the reaction. The higher K_m_ value of Fe_3_O_4_NPs with H_2_O_2_ was consistent with the reported result that Fe_3_O_4_NPs needs a higher H_2_O_2_ concentration than Fe_3_O_4_@LNPs to obtain the comparable V_m_ [11]. Table 1 shows Fe_3_O_4_@LNPs possessed a comparable or stronger affinity to TMB or H_2_O_2_, compared to Fe_3_O_4_ NPs prepared by other methods [36,37,38,39]. Conclusively, the metal nanoparticles loaded on lignin nanoparticles improved its dispersion stability in solution and eventually increased its catalytic capacity. 

### 3.7. The Colorimetric Detection of H_2_O_2_

**Figure 10:**
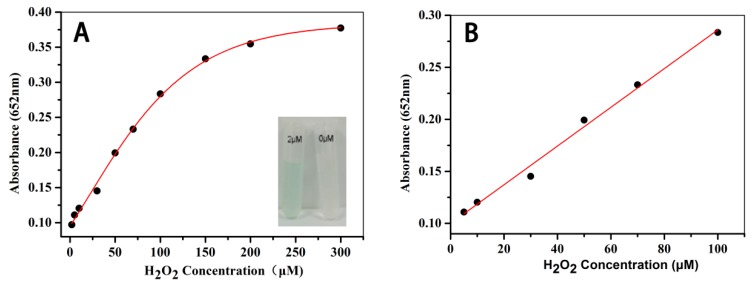
The dose-response curve for H_2_O_2_ detection (**A**), the inset: the colored products of different concentration of H_2_O_2_ (2 μM and 0 μM); the liner correlation between absorbance and H_2_O_2_ concentration (**B**).

Because the inherent peroxidase-like activity of Fe_3_O_4_@LNPs was H_2_O_2_ concentration dependent, a colorimetric method was designed to detect H_2_O_2_. Under the optimal conditions, in a total reaction volume of 3 mL, 264 μg/mL Fe_3_O_4_@LNPs and 4 mM TMB mixed with various concentrations of H_2_O_2_ in a CPBS buffer (pH = 3.0), the reaction was completed at 50 °C for 30 min. It was demonstrated in Figure 10A the solution color and the absorbance were correlated with H_2_O_2_ concentration. For example, the solution color became darker and the absorbance increased, as the concentration of H_2_O_2_ increased. The LOD of H_2_O_2_ concentration corresponding to the color variation was as low as 2 μM (Figure 10A, inset). When the concentration of H_2_O_2_ was less than 100 μM, there was a linear relationship between the absorbance at 652 nm and the H_2_O_2_ concentration. Conclusively, there was the possibility of the rapid detection of H_2_O_2_ by the naked eye or a UV-visible spectrophotometer when the Fe_3_O_4_@LNPs was applied.

The linear relationship between the absorbance at 652 nm and the H_2_O_2_ concentration was identified when the concentration of H_2_O_2_ ranged from 5 to 100 μM (Figure 10B). The linear regression equation was A_652nm_ = 0.09993 + 0.00186C (where C is the concentration of H_2_O_2_), and the correlation coefficient (R^2^) is 0.99077 (*n* = 3). The LODwas 2 × 10^−6^M, which is lower than the counterpart of Fe_3_O_4_ NPs (3 × 10^−6^ M) [39].

## 4. Conclusion

Fe_3_O_4_@LNPs were prepared from the purified Spruce lignin using the self-assembly method. TEM and DLS analysis indicated that when the initial concentration of lignin solution was 1.0 mg/mL, the homogenous Fe_3_O_4_@LNPs was obtained, and the average size was 152.8 nm. The catalytic ability of Fe_3_O_4_@LNPs was optimized with the reaction time, pH, and temperature. Fe_3_O_4_@LNPs exhibited better peroxidase mimic activity than Fe_3_O_4_ NPs due to its more stable dispersion in the reaction system. The catalytic activity of Fe_3_O_4_@LNPs allows its colorimetric detection of H_2_O_2_ at 2 μM limitation of concentration.

## Figures and Tables

**Table 1:** Comparisons of K_m_ and V_m_ among Fe_3_O_4_@LNPs, Fe_3_O_4_ NPs, and HRP.

Catalyst	Substrate	K_m_/mM	V_m_/10^−8^ Ms^−1^	Reference
Fe_3_O_4_@LNPs	TMB	0.51	1.03	This work
Fe_3_O_4_@LNPs	H_2_O_2_	5.30	0.96	This work
Fe_3_O_4_ NPs	TMB	0.01	3.44	[11]
Fe_3_O_4_ NPs	H_2_O_2_	154	9.78	[11]
HRP	TMB	0.43	10.00	[11]
HRP	H_2_O_2_	3.70	8.71	[11]
His-Fe_3_O_4_	H_2_O_2_	37.99	-	[37]
Ala-Fe_3_O_4_	H_2_O_2_	226.60	-	[37]
P-Fe_3_O_4_	TMB	0.44	-	[36]
CDs-Fe_3_O_4_	H_2_O_2_	56.97	-	[38]
GO-Fe_3_O_4_	H_2_O_2_	305.00	1.01	[40]

Note: His-Fe_3_O_4_: Histidine modified Fe_3_O_4_; Ala-Fe_3_O_4_: Alanine modified Fe_3_O_4_: Not given; P-Fe_3_O_4_: Porphyrin modified Fe_3_O_4_; CDs-Fe_3_O_4_: Carbon dots modified Fe_3_O_4_; GO-Fe_3_O_4_: Graphene oxide-based Fe_2_O_3_ hybrid.
